# Children Centered Care: child and parent perspectives on a multi-faceted concept for magnetic resonance imaging without anesthesia – a survey

**DOI:** 10.1007/s00247-024-06111-3

**Published:** 2024-12-11

**Authors:** Stine Bjerrum Runge, Helle Precht, Ib Erik Jensen, Kim Jensen, Tine Abildgaard Johannesen, Malene Roland Vils Pedersen, Nicolaj Lyhne Christensen

**Affiliations:** 1 Department of Radiology, Lillebaelt Hospital Kolding, Sygehusvej 24, 6000 Kolding, Denmark; 2 Department of Radiology, Lillebaelt Hospital Vejle, Vejle, Denmark; 3 Department of Regional Health Research, Odense, Denmark; 4 Health Sciences Research Center, UCL University College, Odense, Denmark; 5 Department of Radiology, Odense University Hospital, Odense, Denmark; 6 Progardia, Middelfart, Denmark

**Keywords:** Anesthesia, Anxiety, Children, Comfort, Magnetic resonance imaging, Parents, Survey, Visual analogue scale

## Abstract

**Background:**

Anxiety-provoking healthcare procedures require specific child-friendly approaches. Magnetic resonance imaging (MRI) can cause anxiety for children and general anesthesia (GA) is often used. We developed and tested a multi-faceted child-friendly concept, Children Centered Care, for MRI of children without GA.

**Objective:**

To investigate children’s and parents’ individual experiences with the concept using a survey. The main aim was to evaluate comfort for children and sense of security for parents during unsedated MRI.

**Materials and methods:**

In this prospective study of 265 children aged 4–10 years enrolled in 2016 and 2017, the Children Centered Care concept is compared to a standard setup. The concept included an interactive app, trained pediatric radiographers, a children’s lounge with a toy-scanner, and a child-friendly multimedia environment in the scanner room. A 25-item survey was used including a mix of open and closed questions, free text, and a visual analogue scale to evaluate self-reported child comfort.

**Results:**

A total of 154 children were included in the Children Centered Care group and 111 in the standard group. Overall, the mean age was 8.5 years (range 4.0–10.9 years).

With Children Centered Care, child comfort increased (88% vs. 77%), *P* = 0.02. The app and toy-scanner were popular among children. More parents felt “very much” prepared (80% vs. 57%), *P* < 0.01, and “very much” secure (92% vs. 79%), *P* < 0.01.

**Conclusion:**

With the use of a multi-faceted, child-friendly concept, MRI without GA is a feasible first choice for children aged 4–10 years, with high levels of comfort for children and parents.

**Graphical Abstract:**

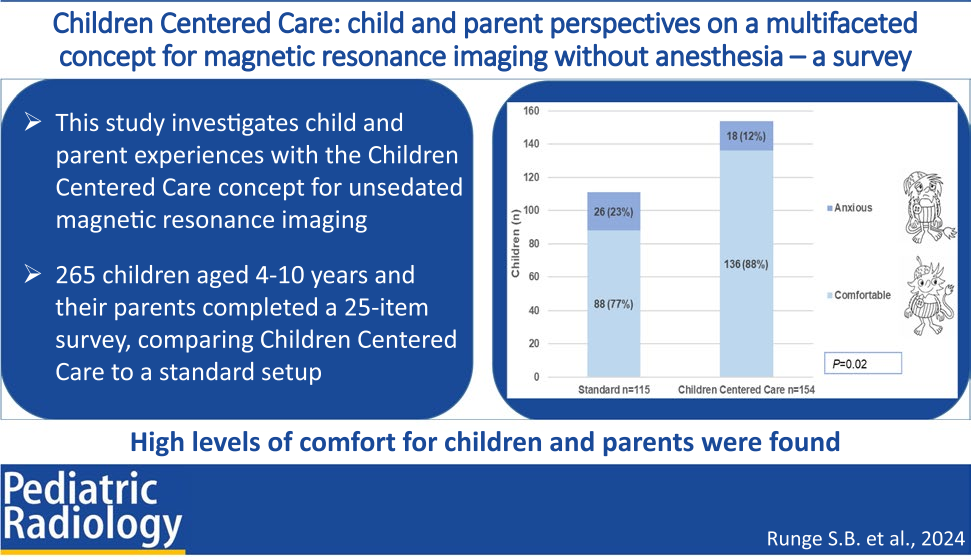

## Introduction

The approach to children in healthcare has been evolving over the past decades, and still is. Children’s rights and competences are in focus, and new setups aim to reduce anxiety and the use of restraint and to increase children’s comfort and autonomy. Experiences from one procedure can influence a child’s coping with the next. Thus, optimizing conditions for children should be of high priority in all aspects of up-to-date pediatric care.

Magnetic resonance imaging (MRI) is a highly favorable diagnostic modality in children, offering images with excellent soft tissue contrast without radiation exposure. However, the unfamiliar environment, loud noises, confined space, and the need to remain still for a longer period of time can be stressful and frightening to children [1–3]. To overcome this, general anesthesia (GA) is often used. While serious medical complications are rare, GA requires special equipment and anesthetics staff, thus increasing costs and waiting times [4–6]. Importantly, GA too is frightening for many children, and the use of physical restraint during induction is not uncommon [7–9].

Various child-friendly approaches to MRI have been reported [10, 11]. Some papers report an effect of single interventions to improve child comfort during MRI. We wanted to go “all-in” to create a comprehensive, child-friendly setup for unsedated MRI and improve children’s comfort.

We developed a multi-faceted concept for children aged 4–10 years, Children Centered Care. It included an interactive app, trained pediatric radiographers, a children’s lounge with a toy MRI scanner, and a child-friendly multimedia environment in the MRI room. The markedly reduced need for anesthesia to 5% for children aged 4–6 years with the Children Centered Care concept and its effects on image quality and waiting times together with a cost–benefit analysis are reported elsewhere [12].

Evaluating child comfort is challenging, as it cannot be measured objectively. Methods include qualitative interviews, specific anxiety scales, Likert scales, and visual analogue scales [13–19]. Some use an external observer and some ask the parents, while others rely on the children themselves. We wanted to ask the children directly and developed a visual analogue scale for our survey. The present study evaluates children’s and parents’ experiences with the Children Centered Care concept, with the main aim to assess child comfort during MRI.

## Materials and methods

**Fig. 1:**
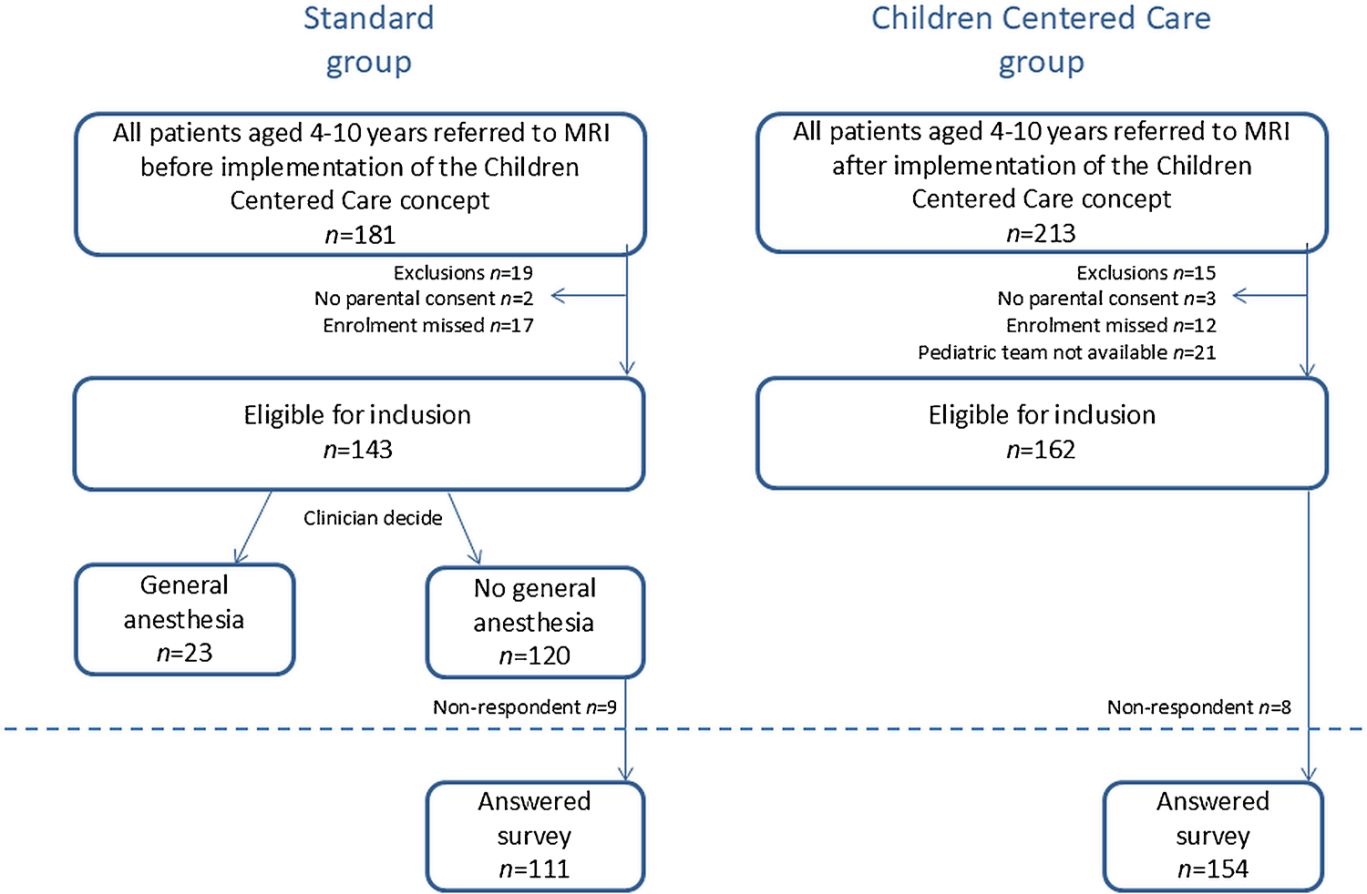
Flowchart for inclusion and exclusion of patients before and after the implementation of the Children Centered Care concept. Study population below the *dotted line*. MRI magnetic resonance imaging

The multi-faceted, child-friendly concept Children Centered Care was developed for children aged 4–10 years. Development took 18 months from the time funding was granted to the first child being scanned. In this prospective study, we compared survey results before and after implementation of the Children Centered Care concept in a Danish secondary center. All children aged 4–10 years referred to our department for MRI from February to September 2016 and 2017, respectively, were screened for inclusion into the standard and Children Centered Care groups, respectively (Fig. 1). A priori exclusion criteria were absence of parental consent, mental disability, or cerebral palsy at Gross Motor Function Classification System level III-V. Only the first visit of a child during each period was included in the study.

MRI scans included head, neck, spine, pelvis, abdomen, small intestine, extremities, and angiography (Table 1). A Philips Ingenia 1.5-T MRI scanner (Philips Healthcare, Best, Netherlands) was used. Standard protocols for our department were used and were unchanged for the Children Centered Care concept. The most common examination was brain MRI. Our standard brain protocol contains transverse T2-weighted Multivane-XD, sagittal T2-weighted fluid-attenuated inversion recovery (FLAIR) BRAIN VIEW 3-dimensional (D), and transverse diffusion-weighted imaging sequences. The 3-D FLAIR sequence is highly susceptible to motion and was replaced by scanning in all three planes when necessary to maintain diagnostic image quality. For scanning with intravenous contrast, relevant T1 and T2 sequences were added. For all scans, extra sequences could be added by the radiologist when relevant. A senior radiologist reviewed and approved the images before the exam was concluded. The total time in the MRI room (time in MRI room) and the start and finish of the scan (net scan time) were registered.

**Table 1:** Patient characteristics

	Standard		Children Centered Care		*P*-value
	*n* = 111		*n* = 154		
Sex					0.43
Girls	58	52%	88	57%	
Boys	53	48%	66	43%	
Age (years)					
4	1	1%	9	6%	
5	3	3%	9	6%	
6	13	12%	20	13%	
7	19	17%	22	14%	
8	22	20%	26	17%	
9	28	25%	34	22%	
10	25	23%	34	22%	
Type					0.17
Inpatients	17	15%	15	10%	
Outpatients	94	85%	139	90%	
Referring clinician					0.45
Neuropediatric	50	45%	78	51%	
Other pediatric	4	4%	2	1%	
Orthopedic surgery	53	48%	74	48%	
Abdominal surgery	3	3%	0	0%	
Oncology	1	1%	0	0%	
Anatomical region					0.33
Head, neck, spine	58	52%	83	54%	
Extremities, pelvis	46	41%	67	44%	
Abdomen, thorax	7	6%	4	3%	
Previous MRI					0.48
Yes	26	23%	42	27%	
No	84	76%	112	73%	
N/A	1	1%	0	0%	
Blood test and/or intravenous access on scan day					0.02
Yes	24	22%	17	11%	
No	87	78%	137	89%	

*MRI* magnetic resonance imaging

### Standard group

In the standard group, children were included from February to September 2016 and scanned using our standard setup (Fig. 1). Pre-procedural information was given in the appointment letter and non-systematically by the referring department. Children were scanned by random MRI radiographers with no specific pediatric communication training. Music was available in headphones during the scan. Children were referred directly to MRI in GA, if the referring clinician expected GA was necessary. Children scanned in GA were not able to answer the survey. Three children failed their MRI due to anxiety and did not answer the survey. They were included in the analysis of child comfort categorized as “anxious.”

### Children Centered Care group

The entire Children Centered Care concept was implemented and tested from October 2016 to January 2017 in between data collection of the standard and Children Centered Care groups. In the Children Centered Care group, children were included from February to September 2017 (Fig. 1). In this period, *all* children were scanned without GA using the Children Centered Care concept. If a diagnostic MRI was not completed, an examination in GA was offered 2 days later.

**Fig. 2:**
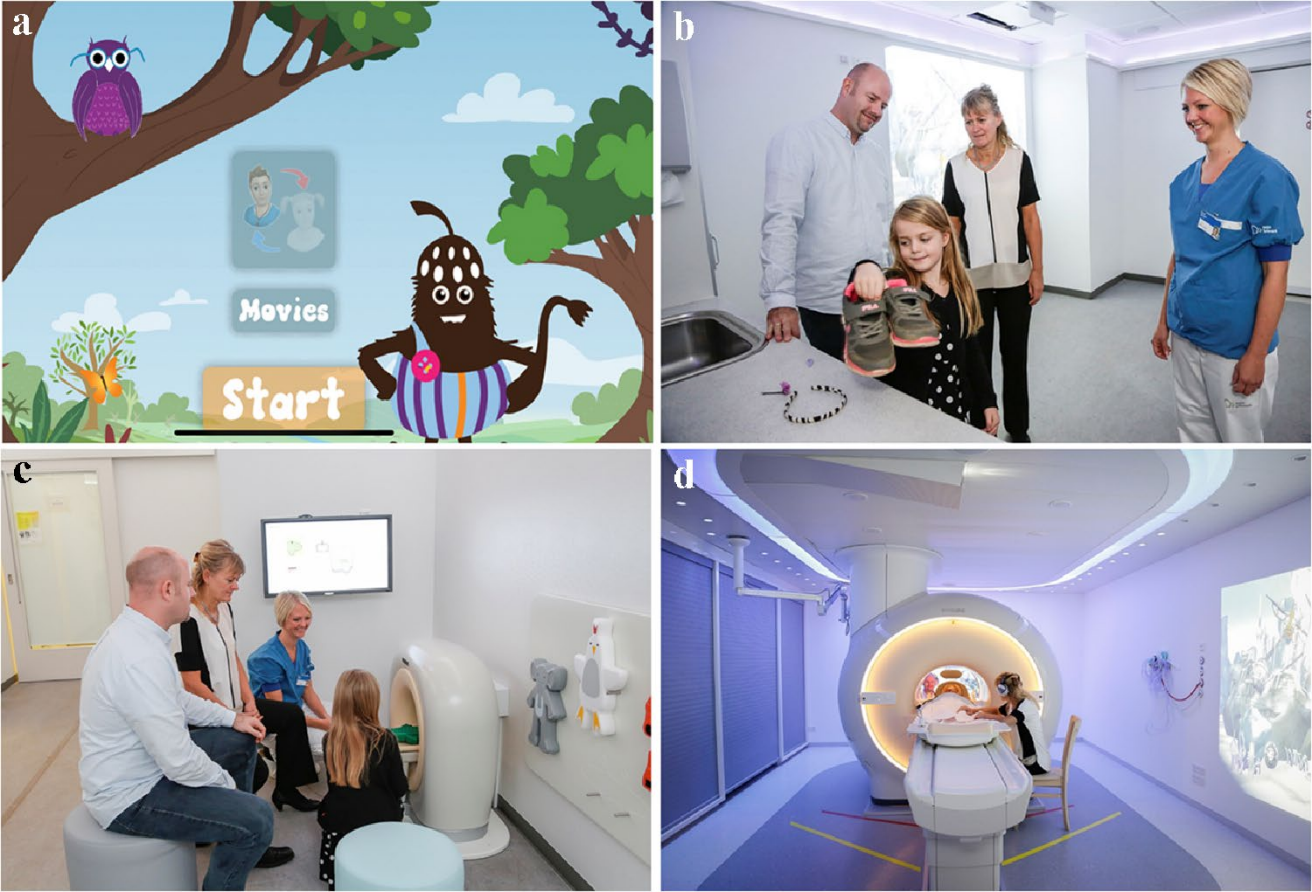
The four elements of the Children Centered Care concept for magnetic resonance imaging. **a** An interactive app prepares the child through gamification. **b** The child is met by trained pediatric radiographers who support the child and their parents through the visit. **c** In the children’s lounge, the toy-scanner provides an opportunity for the radiographer to interact playfully with the child. **d** Positive distraction with different multimedia solutions creates a comfortable, child-friendly environment in the scanner room and during the scan. Staged photos for illustration

The Children Centered Care concept includes four main elements (Fig. 2):

1. An interactive app with the animated character “Rumble”
2. A team of radiographers trained in pediatric communication
3. A children’s lounge with a toy-scanner accessed before the MRI
4. A child-friendly MRI room with coordinated lights and movie themes

To qualify our concept further, we collaborated with two innovation consultants from the Health Innovation Centre of Southern Denmark, an organization specializing in developing innovative solutions at hospitals through user involvement methods. They mapped the complete patient journey from referral to finished exam. Valuable insights were obtained from observations of five children going to MRI equipped with a mounted camera, recording their way from the hospital lobby to the MRI room, finishing with a comprehensive interview. Also, eight children, scanned within the past 2 months, were invited to focus group interviews sharing their thoughts and experiences. Numerous detailed insights from the field research were used developing the app, designing the children’s lounge and educating the radiographers. Wayfinding in the hospital and the metal checklist were made more child-friendly. Some suggestions were small and practical, like placing a footstool at the reception desk to enable the child to interact with the receptionist.

#### App

The app was developed specifically for our department with 3-D animations of the actual rooms in collaboration with a third-party app developer (English version “Rumble in MRI” free in App Store and Google Play Store, OddLabs, Denmark). The child is guided through the entire MRI procedure by “Rumble,” an animated forest troll, well-known from the hospital’s pediatric department. Elements of gamification were added to motivate the child and reinforce learning, i.e., by helping to remove metal items. The sounds of an MRI scanner are demonstrated. The toy-scanner and characters from the children’s lounge are introduced, as well as the movie themes in the MRI room.

#### Children’s lounge and toy-scanner

The children’s lounge is a dedicated room where the radiographer prepares the child for the MRI in calm, child-friendly surroundings. The child and radiographer play together, scanning four physical characters, already introduced in the app, in a toy-size MRI scanner (“Kitten scanner package,” Philips Electronics, Koninklijke, Netherlands). As the child places the character in the toy-scanner, a TV screen is playing a small cartoon ending with a diagnosis, e.g., the crocodile Chris who has stomach pain turns out to have swallowed a pirate. The session was scheduled for 15 min.

#### Pediatric team

We included five radiographers in the pediatric team, selecting highly motivated staff with pediatric experience. They received specific training during three all-day workshops led by two pediatric nurses specializing in communication training based on the Calgary Cambridge Guide [20].

Insights from the user involvement process (mounted camera, focus groups) were presented by the innovation consultants. The pediatric nurses held a 2-h didactive session and supervised 5 h of roleplay. Video recordings of previous MRI sessions were used to give feedback to the radiographers. Know-how and tips from the workshops were collected in a written guide for radiographers (“The Good Welcome. Making children feel comfortable”).

#### Child-friendly environment in MRI room

From the children’s lounge, the child proceeds to the preparation room. Here the child removes metal items as learned in the app.

The child chooses among ten movie themes projected onto the walls of both the preparation and MRI room while a customized light setup creates an ambient atmosphere (“Ambient Experience,” Philips Electronics). Inside the MRI room itself, sounds and music are accompanying the movie themes, stimulating the child to enter. The parent is positioned on a chair next to the child and is able to touch the child during the scan. A mirror on the base coil (where applicable) allows the child to see the parent during the scan, as well as a supplementary TV screen behind the scanner.

### Online survey

We developed a 25-item survey with a focus on children’s and parents’ individual experiences during MRI without GA. The questions were developed with the aid of the innovation consultants and based on advice from an experienced pediatric quality improvement nurse.

The child and one parent were asked to participate in an online survey, using an electronic device (iPad, Apple Inc., Cupertino, CA) handed to the child and parent directly after the MRI scan. All radiographers were blinded to all responses. Participation was voluntary.

SurveyXact (Ramboll Management Consulting, Aarhus, Denmark) was used to devise the survey and the text was in Danish. Only essential questions were included to increase the response rate. Questions included a mix of closed questions and scale responses. Participants could skip questions not applicable to them. The first part of the survey included 16 questions for the parent and the last part nine questions for the child.

**Fig. 3:**
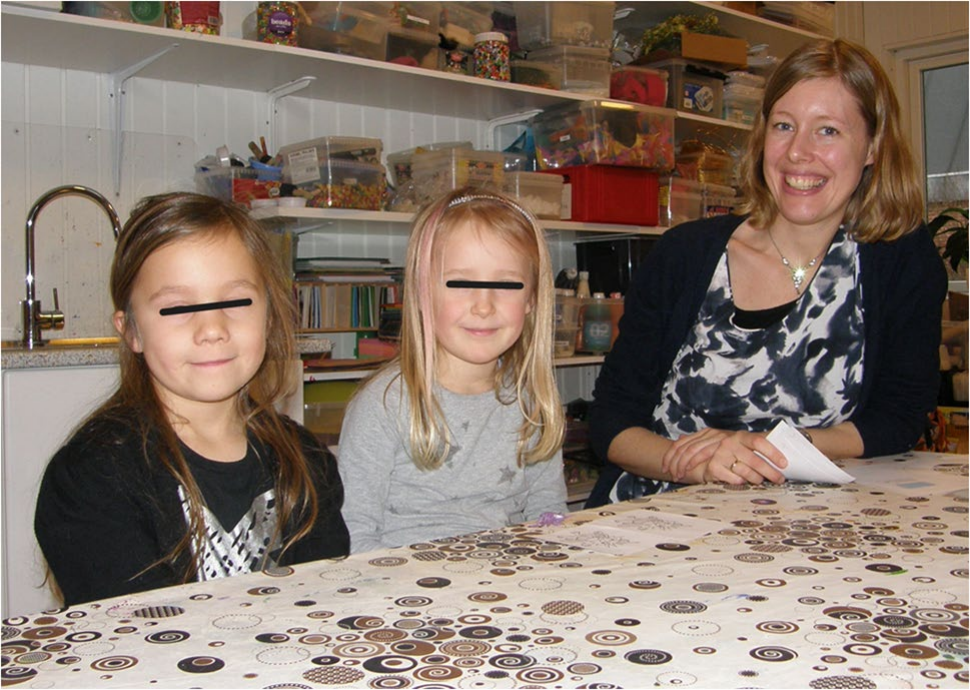
Pilot testing the visual analogue scale for evaluating comfort of children during magnetic resonance imaging in a Danish kindergarten (2016). Published with written consent from parents. Photo: Lotte Blanner

**Fig. 4:**
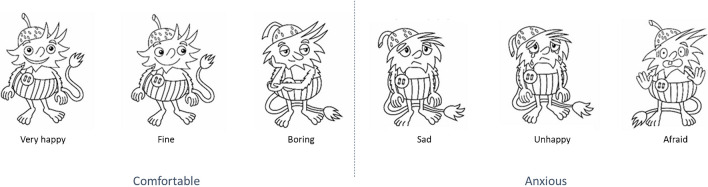
The visual analogue scale developed using the animated character Rumble, including the six most relevant emotional states of children during magnetic resonance imaging

To evaluate child comfort, we included a visual scale in the survey using “Rumble,” the animated character also used in the app. It was pilot tested in spring 2016 in a Danish kindergarten with 12 children aged 4 years to 5 years (Fig. 3) with aid from the innovation consultant. The children helped to adjust and validate ten animated emotional figures, by telling what they thought each Rumble figure was feeling like when placed in a paper model of an MRI scanner. After adjustment by the illustrator, the figures were presented to a focus group of children aged 5 years to 10 years, who had had an MRI scan at our department within the past 2 months. Based on their feedback, six figures showing the most relevant emotional states for children during MRI were selected, and titles (in Danish) were added and categorized as “comfortable” or “anxious” (Fig. 4). The Rumble figures were used in the online survey to measure child comfort in the waiting room and during the scan, respectively. Categories “Don’t know” and “Wasn’t in lounge/scanner” were added.

The complete survey was tested by five children and parents before data collection began.

### Statistics

Data are expressed as numbers and percentages. Numerical values are expressed as median and interquartile ranges (IQR). Associations between groups were tested using the chi-square test or Fisher’s exact test if appropriate. Logistic regression was performed to assess the association between scan time and comfort level. We used Excel version 2403 (Microsoft, Redmond, WA) and STATA/SE 18.0 (StataCorp LP, College Station, TX). A value of *P* < 0.05 for a two-tailed test was considered statistically significant.

## Results

Results are shown in Tables 1, 2, 3, 4, and 5. We included 154 children in the Children Centered Care group and 111 in the standard group. Six and eight children in the standard and Children Centered Care groups, respectively, did not answer the survey and were excluded, yielding an overall response rate of 95%.

**Table 2:** Children’s experiences with the app and toy-scanner

	Children Centered Care	
Did you like to use the app? *n* = 95		
Yes, a lot	69	73%
Yes, a little	24	25%
No, not that much	2	2%
No, not at all	0	0%
Don’t know	0	0%
Did the app “Med Rumle i MR” show well what happens when you have an MRI scan? *n* = 95		
Yes	90	95%
No	2	2%
Don’t know	3	3%
Did you like to use the toy-scanner *n* = 137		
Yes, a lot	102	74%
Yes, a little	27	20%
No, not that much	5	4%
No, not at all	0	0%
Don’t know	3	2%
Did the toy-scanner show well what happens when you have an MRI scan? *n* = 137		
Yes	124	91%
No	6	4%
Don’t know	7	5%

“*Med Rumle i MR*” “Rumble in MRI,” *MRI* magnetic resonance imaging

**Table 3:** Children’s experiences in the waiting room/children’s lounge. Selected questions. Bold, comfortable. Italic, anxious

	Standard Waiting room		Children Centered Care Children’s lounge	
	*n* = 111		*n* = 154	
How did you feel when you were in the waiting room/children’s lounge before your MRI scan? Please choose the character that best shows how you felt				
**Very happy**	**22**	**20%**	**71**	**46%**
**Fine**	**49**	**44%**	**56**	**36%**
**Boring**	**22**	**20%**	**8**	**5%**
*Upset*	*1*	*1%*	*3*	*2%*
*Sad*	*2*	*2%*	*3*	*2%*
*Afraid*	*8*	*7%*	*9*	*6%*
Wasn’t in waiting room/lounge	4	4%	2	1%
Don’t know	3	3%	2	1%
Would you like to have an MRI scan again?				
Yes	38	34%	74	48%
No	47	42%	45	29%
Don’t know	26	23%	33	21%
Missing	0	0%	2	1%

*MRI* magnetic resonance imaging

**Table 4:** Emotional states during magnetic resonance imaging comparing the standard and Children Centered Care groups. Self-reported data using a visual analogue scale. Bold, comfortable. Italic, anxious

	Standard		Children Centered Care	
	*n* = 114		*n* = 154	
How did you feel, as you were inside the MRI scanner? Please choose the figure that best shows how you felt				
**Very happy**	**25**	**22%**	**44**	**29%**
**Fine**	**46**	**40%**	**75**	**49%**
**Boring**	**17**	**15%**	**17**	**11%**
*Upset*	*3*	*3%*	*1*	*1%*
*Sad*	*8*	*7%*	*7*	*5%*
*Afraid*	*10*	*9%*	*9*	*6%*
**Wasn’t in scanner due to anxiety**	**2**	**2%**	**1**	**1%**
**Failed due to anxiety, did not answer survey**	**3**^a^	**3%**	**0**	**0%**
Don’t know	0	0%	0	0%

*MRI* magnetic resonance imaging

^a^In the specific analysis of child comfort, three children who failed due to anxiety and did not answer the survey were included in the analysis categorized as “anxious”

**Table 5:** Parents’ experiences during their child’s magnetic resonance imaging procedure

	Standard		Children Centered Care		*P*-value
	*n* = 111		*n* = 154		
How did you, as a parent, experience the waiting room/children’s lounge? (you can choose more than one answer)					
Informative	7	6%	75	49%	
Child-friendly	16	14%	134	87%	
Safe	23	21%	73	47%	
Neutral	54	49%	11	7%	
Clinical	31	28%	2	1%	
Stressful	8	7%	1	1%	
Other	8	7%	4	3%	
Did you feel well prepared before your child’s MRI scan? Please consider both the information you got before you arrived and at the x-ray department					
Very much	63	57%	123	80%	< 0.01
Somewhat	38	34%	27	18%	
Not so much	7	6%	3	2%	
Not at all	2	2%	0	0%	
Don’t know	1	1%	1	1%	
Did you feel secure during the process of your child’s MRI scan?					
Very much	88	79%	141	92%	< 0.01
Somewhat	22	20%	12	8%	
Not so much	0	0%	0	0%	
Not at all	0	0%	0	0%	
Don’t know	1	1%	1	1%	

*MRI* magnetic resonance imaging

Table 1 shows patients’ characteristics. The mean age in the Children Centered Care group (8.7 years) was similar to that in the standard group (8.9 years), *P*-value 0.19. The age range was 4.9–10.9 years in the standard group and 4.0–10.9 years in the Children Centered Care group. In the analysis of child comfort (115 children included), children aged 4 years and 5 years comprised a smaller proportion in the standard group (4%) compared to the Children Centered Care group (12%).

Inpatients comprised a smaller share in the Children Centered Care group than in the standard group, but the difference was not significant. Previous experience with MRI was equally frequent among children in the Children Centered Care and standard groups. Fewer children got a blood test and/or intravenous access on the scan day in the Children Centered Care group, *P* = 0.01.

The time in the MRI room was 33 min (IQR 26.5; 45) vs. 30 min (IQR 24.5; 42.5) in the standard group and the Children Centered Care group, respectively (*P* = 0.25). The net scan time was 25 min (IQR 19; 35.5) vs. 25 min (IQR 18; 35) in the standard group and the Children Centered Care group, respectively (*P* = 0.72).

In the Children Centered Care group, 77% (119/154) of parents received information about the Rumble app and 72% (111/154) reported that their child used the app. Table 2 shows how children rated the app and toy-scanner. Of the children who confirmed having used the app, 98% (93/95) liked to use it. The toy-scanner was used by 90% (137/154) of children in the Children Centered Care group, they reported, and 94% (129/137) liked to use it.

**Fig. 5:**
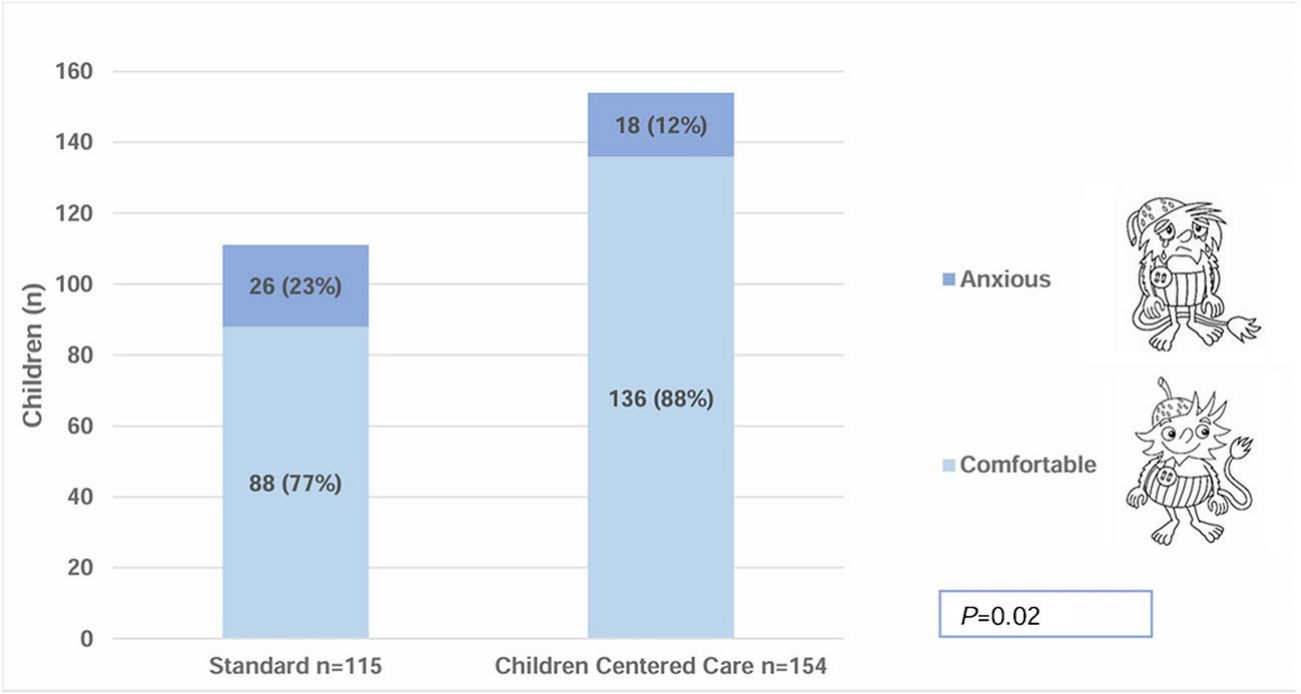
Child comfort during magnetic resonance imaging comparing the standard and Children Centered Care groups. Self-reported data using a visual analogue scale after categorization into “comfortable” or “anxious”

Tables 3 and 4 and Fig. 5 show children’s experiences with the standard setup and the Children Centered Care concept. In the Children Centered Care group, 88% (136/154) of children felt comfortable during their unsedated MRI, compared to 77% (88/115) in the standard setup, *P* = 0.02.

Child comfort was not associated with net scan time using logistic regression analysis, OR = 0.98, 95% CI [0.95, 1.01] in the standard group (*P* = 0.22) and OR = 1.0, 95% CI [0.97, 1.03] in the Children Centered Care group (*P* = 0.95).

The success rate of completing a diagnostic MRI was 95% (108/114) among children referred to MRI without GA in the standard setup. In the Children Centered Care concept, all children attempted MRI without GA and 98% (151/154) succeeded. The success rate for the subgroup of children aged 4–6 years has been reported and discussed elsewhere [12].

Six children in the standard group and three in the Children Centered Care group did not complete their MRI. Eight of these fails were due to anxiety, while one child in the Children Centered Care group was in pain (as noted by the radiographer). None of the children in the standard group had blood tests or intravenous access on the scan day (data missing for the three non-responders). In the Children Centered Care group, all three children with failed exams had either blood tests or intravenous access.

In the Children Centered Care group, 48% (74/154) reported that they would like to have an MRI scan again, compared to 34% (38/111) in the standard group.

Table 5 shows parents’ experiences. The children’s lounge was experienced as child-friendly (87%), informative (49%), and safe (47%) by parents in the Children Centered Care group. Only 14% of parents found the waiting room in the standard setup child-friendly, *P* < 0.01. With Children Centered Care, more parents felt “very much” prepared for their child’s MRI scan (80% vs. 57%), *P* < 0.01. The proportion of parents who felt “very much” secure during the process of their child’s MRI scan increased from 79 to 92%, *P* < 0.01.

## Discussion

This survey included 265 children aged 4–10 years and their parents with a 95% response rate. With Children Centered Care, our multi-faceted concept, 88% of children reported to feel comfortable during their unsedated MRI. This proportion was even higher than in the standard setup, where only selected children expected to succeed had unsedated MRI (77%), *P* = 0.02. Children liked to use the app and toy-scanner. Parents felt more prepared and secure during their child’s MRI with the Children Centered Care concept.

Competent care of children in healthcare requires specific approaches aimed at the relevant age group, especially for anxiety-provoking or painful procedures. To enable children to cooperate during unsedated MRI, the following elements should be considered when designing a setup: information, recognizability, motivation, child-friendly communication, and positive distraction [21, 22]. Individual children may benefit from some elements more than others. In some studies, the youngest children were found to benefit the most from various child-friendly features [23, 24].

The finding that 88% of children felt comfortable with the Children Centered Care concept is very satisfactory, as the study population included all children aged 4–10 years referred to an MRI in the intervention period, including inpatients, with few exclusion criteria and no further selection based on child age or maturity. Also, MRI was completed without GA or sedation by 98% of children. We believe that the multi-faceted approach is the basis for this, each of the four elements contributing to meet the child’s needs when placed in a demanding situation in a foreign environment, as discussed below. Likely, the interventions work in synergy to empower the child to successfully complete the scan and feel comfortable. The effect of individual elements cannot be evaluated from this study.

When planning the project, we only found a few other studies of children’s experiences with MRI, especially involving children aged 4–6 years. In this age group, the use of anesthesia is common [25–27], which makes the question of comfort during the scan irrelevant. Some studies use healthy volunteers assessing anticipated anxiety for an imagined or simulated MRI scan which may introduce substantial selection bias with results not applicable to a clinical setting [28, 29]. Others introduce an intervention and measure anxiety before and after the MRI, but as no control group is included, the effect of the intervention cannot be assessed [16, 19].

### App

The app is central in the Children Centered Care concept and has several obvious advantages. Firstly, it delivers age-appropriate information essential for the preparation of the child. Also, it is used at home in familiar surroundings and without the time limits of a hospital schedule. The gamification elements motivate the child to explore the app in a familiar way known from leisure gaming. It connects individual elements of the Children Centered Care concept and introduces them to the child.

“Rumble” the troll character, who many children know from the pediatric department at our hospital, was used in the app and in physical wall decorations in the MRI section. Thereby, recognizability and continuity during the process of an MRI were secured, optimizing the app for local use. Using “Rumble” may have made the app less generic though, as other hospitals might have their own character/mascot. Also, “Rumble” might not suit the taste of all children world-wide. In the future, app developers could consider using exchangeable or more generic characters. The app was liked by 98% of the children who used it. Among enrolled children, 77% had received information of the app, and of these, 95% had used it. Thus, parents and children are well-motivated to use an app for at-home preparation. Ensuring that all families are informed of the app should be of high priority in the future.

### Children’s lounge and toy-scanner

Preparation in the children’s lounge was done in just 15 min, leaving the MRI scanner available to other patients. In practice, the toy-scanner acts as a great tool for the radiographer to connect with the child through play. Having used the app at home, children are familiar with the situation and seem empowered to take a lead at the toy-scanner, motivated to scan the four characters they know from the app to help them get a diagnosis. Parents may naturally withdraw a bit, leaving space for the radiographer to lead the dialogue with the child.

Using the toy-scanner, the need for an actual mock session is eliminated, which is otherwise a common approach in preparation of children for MRI [24, 25, 30]. Importantly, a mock session can be time-consuming and often requires an extra visit to the hospital. Preparation with the toy-scanner saves time for families as well as staff and simplifies patient flow. Morel et al. evaluated the impact of a *teddy bear-scale mock MRI scanner* on the anxiety level of 91 children aged 4–16 years using a visual analogue scale of stress-level from 0 to 100 [18]. They found that children’s anxiety levels were significantly lower after a mock MRI than before. This was not the case in the control group. Actual test values and reductions were not reported. Overall, these findings confirm that a mock session with a toy-scale scanner can reduce anxiety.

Nearly all children in the Children Centered Care group used the toy-scanner (90%). Thus, this form of preparation worked well in practice and was easier to implement than the app.

### Pediatric team

Highly motivated radiographers were specifically recruited for the Children Centered Care concept. With our training program, skills of communication and cooperation with children were further improved. By selecting a smaller group, individual radiographers continuously gained more experience and know-how. The size of the team must fit the desired capacity and be larger if an out-of-hours service is needed.

Our study indicates that trained radiographers may be as effective as child life specialists, with the advantages of not needing additional staff. In our experience, the radiographer’s approach towards the child is very important. Castro et al. inspected the effect of adding patient-centered communication to preparation with an MRI toy-scanner including 30 children aged 4–10 years in each group [31]. Decreased anxiety was found when the preparation included patient-centered communication, as measured on a 5-point facial image scale.

Establishing a pediatric team is affordable and readily accessible compared to the technical solutions. It is thus a great place to start, when working to improve the pediatric patient’s experience in MRI.

### Child-friendly environment in MRI room

A hospital environment can be frightening and intimidating for a child, not least when it includes large, foreign-looking technical equipment such as an MRI scanner. Presenting the MRI scanner to the child in the app and children’s lounge is likely to already make it more familiar. We believe that child comfort was increased even further in the appealing atmosphere created with the ambient lighting, sound, and movie themes in the scanner room. During the scan, the movies create positive distraction, helping the child to stay comfortable and remain still. The light- and movie theme is selected by the child. Giving children choices in healthcare is considered important and a part of best practice [32].

Some recent studies evaluate the effect on children’s anxiety after adding an additional intervention to an existing multi-faceted, child-friendly setup. Results cannot be compared directly between different studies including ours, due to different anxiety measures.

In their randomized-controlled trial of 122 children aged 3–7 years, Fletcher et al. added a *mock session* to *home-based preparation* materials and training with a *child life specialist* and found overall similar PedsQL VAS scores between groups. However, lower self-reported fear and parent-reported sadness were found in the play-based setting using a mock scanner [33].

Ozdemir et al. evaluated the *preparatory video* made by McGlashan et al. together with *child-friendly communication* inspired by Raschle et al. in a randomized-controlled study of 66 children aged 4–15 years and found lower anxiety scores during MRI in the intervention group [16, 22, 34, 35]. Anxiety levels were measured comprehensively with the State-Trait Anxiety Inventory, the Modified Yale Pre-Operative Anxiety Scale, the Children’s Anxiety Meter-State, and the Children’s Fear Scale by children, parents, and professionals. They also found lower state anxiety levels of parents in the intervention group. Results cannot be directly compared to ours, but the findings are in consistency.

Geuens et al. included 82 children aged 4–10 years in a study evaluating a smartphone *app* with educational mini-games for use at home, in a setup including a *child specialist* and a *movie* in the MRI room [24]. Parent-reported anxiety scores were used and reduced anxiety after preparation with the app was found for 4–6-year-olds. Thus, findings confirm that a preparatory app can reduce pre-scan anxiety for young children. Furthermore, with the app, less than 5 min face-to-face preparation by a child specialist was needed, compared to the 30–60 min previously used. The potential for a preparatory app as part of a practically manageable, child-friendly setup was thus confirmed.

Various methods of assessing child comfort are found in the literature, of which some are validated for specific clinical settings or age groups not relevant for this study. The commonly used State-Trait Anxiety Inventory for Children is validated only from the age of 5 and was found too comprehensive for this context [14]. We preferred asking the children themselves, as we found the child’s perceived experience to be most relevant. Feeling comfortable remains subjective.

Visual analogue scales can be used directly by pre-school children. Using a whole-body figure allowed our illustrator to emphasize the different emotional states by using “Rumble’s” body language. It remains uncertain if the scale can be used across cultures.

Parental emotions before and during pediatric radiologic procedures affect the child [21]. Parents request thorough information before the scan to be able to support their child optimally [27, 36, 37]. Thus, parents’ needs should be met in a pediatric setup. In the Children Centered Care concept, all interventions were aimed mainly at children. Still, parental sense of security increased to a very high level. Thus, when seeing their child’s needs taken care of, psychological security is facilitated for parents. Meeting professional, child-friendly staff allows the parent to withdraw somewhat from the responsibility of the scan being successful. This creates space for the radiographer to lead the situation and interact with the child, in a manner known from the “One Voice” approach in pediatric nursing [38].

More parents felt very well prepared, likely via watching the app with their child. The children’s lounge was found child-friendly by almost all parents, while the waiting room of the standard setup was found “neutral” and “clinical.” By fulfilling their needs, the Children Centered Care concept optimizes parents’ impact on their child, as well as their own experience.

### Strengths and weaknesses

This study is large compared to other studies in the field and includes a standard group to compare with. Inpatients and acute patients were included and exclusion criteria were narrow.

However, differences between the Children Centered Care and standard groups are likely to have led to an underestimation of the effect of the Children Centered Care concept. In the standard group, only children expected to be capable of MRI without GA by the referring clinician were included. These were likely less anxious and/or more mature than children referred to GA (and thus not included in this study). Accordingly, the standard group included a smaller proportion of 4– and 5-year-olds, who are likely to be more anxious due to young age. Thus, the selected children in the standard group were more likely to feel comfortable during their MRI. Still, the proportion of comfortable children increased with Children Centered Care and to a very high level.

Randomization to MRI without GA in the standard setup would not have been ethically safe nor feasible. The effect of the Children Centered Care concept is likely to have had a larger effect on the younger children, but a subanalysis of children aged 4–6 would not be valid due to too few subjects.

Fewer children in the Children Centered Care group had a blood test or intravenous access on the scan day. Having a painful procedure before the scan may lead to greater anxiety during MRI. During the observations of children going through MRI (mounted camera sessions), it became evident that some children got an intravenous access before the exam “just in case” contrast agent was needed. In the Children Centered Care concept, meticulous care was taken to avoid any intravenous access if not strictly necessary. Also, blood tests were performed after the MRI, not before, as part of this strategy. This might have added somewhat to the effect of the concept, and should be considered in any child-friendly MRI setup. There was a trend towards a smaller proportion of inpatients in the Children Centered Care group. This may be due to logistic challenges of enrolling children with acute illness in the study. Children in severe pain may not be able to cooperate during MRI without GA. Also, an MRI tech from the pediatric team was not always available during out-of-hours service. For some acutely ill children, access to MRI without GA might actually have reduced the waiting time for their scan because fasting and anesthetic staff were not needed.

The range of MRI examinations done was comparable in the standard and Children Centered Care groups, as the scanned anatomical regions and the net scan time did not differ. We found no association between comfort level and scan duration. It seems that if the child is comfortable enough to enter the scanner and initiate the scan, the child is unlikely to become anxious later.

Informing all families of the app proved challenging, as only 77% knew about the app. If all children had received the full concept, the effect is likely to have been larger.

The high response rate of 95% is a strength of this study and was obtained by having the families answer the questionnaire while still in the department. However, the timing of the survey may have influenced the answers by both children and parents [39]. The perception of the experience may change over time. Answering while sitting in the department, and knowing the iPad should be handed back to the staff afterwards, could lead to more positive feedback out of politeness or a wish to stay on good terms with staff. As all data were collected this way though, results are comparable.

We previously found that with the Children Centered Care concept, the need for GA for MRI of children aged 4–6 was reduced to 5% [12]. The present study validates this concept even further. Thus, multi-faceted, child-friendly concepts for unsedated MRI should be strongly encouraged.

Our department is a secondary center not specialized in pediatric care. Future studies should assess the feasibility in tertiary centers, where higher patient flows create even more potential for improvements, while the patient population is more complex and may have special needs.

## Conclusion

With our multi-faceted Children Centered Care concept for MRI without GA of children aged 4–10 years, the proportion of children feeling comfortable during their scan increased compared to the standard setup and to a very high level. Parents felt more prepared for their child’s MRI and their sense of security during the scan increased.

With a multi-faceted, child-friendly concept like Children Centered Care, unsedated MRI can and should be first choice for children from the age of 4, with benefits for children, parents, and healthcare professionals.

## Data Availability

The data that support the findings of this study are not openly available due to reasons of sensitivity and are available from the corresponding author upon reasonable request. Data are located in controlled access data storage at our institution.
